# Serum 25(OH)D Levels in Fragility-Fractured and Non-fractured Patients in a Low Latitude Region

**DOI:** 10.7759/cureus.86928

**Published:** 2025-06-28

**Authors:** Paola Maria Blanco-Pertuz, Rita Magola Sierra-Merlano, Óscar Giovanny Iglesias-Jiménez

**Affiliations:** 1 Internal Medicine, University of Cartagena, Cartagena, COL; 2 Rheumatology, University of Cartagena, Cartagena, COL

**Keywords:** 25-hydroxyvitamin d, bone density, fragility fracture, genetics, skin phototype

## Abstract

Introduction: Vitamin D is produced in nature in two main forms: vitamin D2 (ergocalciferol) and vitamin D3 (cholecalciferol). The measurement of serum 25(OH)D is the most accurate way to determine the amount of vitamin D in an individual. The association between 25(OH)D and fragility fractures (FxF) has been recognized for many years, but its direct role is unclear.

Objectives: This study aimed to assess the association between serum 25(OH)D levels and FxF in a hospital-based population in the Colombian Caribbean (latitude ~10.3°), using a cross-sectional design with prospective data collection, while adjusting for confounders such as age, sex, skin phototype, BMI, grip strength, and comorbidities. Secondary objectives included exploring potential effect modification by skin phototype and describing the prevalence of vitamin D insufficiency in this low-latitude cohort.

Methods: A cross-sectional study with prospective data collection was performed comparing serum 25(OH)D levels between FxF and non-fractured (non-FxF) patients. Logistic regression, adjusted for age and sex, identified clinical characteristics associated with FxF.

Results: Forty patients with FxF were compared with 40 non-FxF participants. Women comprised 89% of the FxF group. The median age in FxF was 73 years (interquartile range (IQR) 62-80) and 75 years in non-FxF (IQR 62-80; p = 0.2037). Phototype III skin was most common and acted as a protective factor against FxF with a crude odds ratio (OR) of 0.29 (95% CI: 0.19-0.84; p = 0.011) and an adjusted OR of 0.27 (95% CI: 0.12-0.89; p = 0.015). No statistically significant differences in 25(OH)D levels were found between groups (FxF: 26.0 + 10.0 ng/mL; non-FxF: 26.9 + 10.3 ng/mL; p = 0.6991).

Conclusions: Both FxF and non-FxF patients showed insufficient 25(OH)D levels, echoing findings from Mendelian randomization studies suggesting no causal link between low vitamin D and FxF. Phototype III was the most common and was found to be protective. Phototypes V and VI were not observed in the study cohort. The inclusion of the hospital-based nature of the cohort may explain the overall vitamin D insufficiency and higher grip strength in the group with FxF. Further studies are required to assess the interaction of genetic and metabolic factors in this context.

## Introduction

Vitamin D is naturally found in two primary forms: vitamin D2 (ergocalciferol), derived from plant sources and fungi, and vitamin D3 (cholecalciferol), which is of animal origin and synthesized in the skin upon exposure to ultraviolet B (UVB) radiation actin on 7-dehydrocholesterol. In humans, vitamin D3 undergoes two hydroxylation steps: first in the liver to form 25-hydroxyvitamin D (25(OH)D), and then in the kidneys to produce the biologically active form 1,25-dihydroxyvitamin D (1,25(OH)2D) [1]. Measurement of serum 25(OH)D is considered the most reliable indicator of an individual’s vitamin D status in both clinical and research contexts.

25(OH)D levels can vary by approximately 25% depending on latitude, altitude, season, dietary intake, and genetic polymorphisms [2]. Variants in genes such as CYP2R1, DHCR7, CYP24A1, and the vitamin D binding protein (GC or DBP) can explain up to 70% of the variation in 25(OH)D levels, especially after UVB exposure [2]. 25(OH)D is related to latitude. People living at higher latitudes experience greater seasonal variability in 25(OH)D levels than those near the equator, a pattern consistently demonstrated in recent large-scale studies [3].

According to international guidelines, 25(OH)D levels are categorized as sufficient (≥ 30 ng/mL), insufficient (20-29 ng/mL), and deficient (≤ 20 ng/mL) [4,5]. 25(OH)D deficiency is associated with impaired bone mineralization, increased parathyroid hormone level, and a higher risk of falls and fragility fractures (FxF) [5].

FxFs are low-energy fractures resulting from a fall from standing height or less that, in healthy individuals, would not usually result in a fracture [6,7]. FxFs are frequently observed in the hip, vertebrae, and radius (Colles fracture) [8]. The lifetime risk of a hip FxF is approximately 14.6% in women and 3.5% in men over the age of 50 [9]. Osteoporosis (OP) significantly increases the risk of subsequent fracture by 2.5 to three times [10] and causes disability in 25% to 75% of cases who had previously been able to walk independently, many of whom lose that ability one year after the event. Recovery often does not restore the prior level of functionality, resulting in reduced quality of life [11].

The importance of 25(OH)D in bone health has been recognized for many years, but its direct role in the FxF relationship is unclear, and research studies are scarce, in contrast to studies of OP and FxF. In a prospective study conducted in the USA to determine the relationship between 25(OH)D and FxF, including non-Hispanic White elderly patients (over 65 years), the multivariate adjusted relative risk (RR) was 0.64 (95% CI: 0.46-0.89) among individuals with serum 25(OH)D values of 18 ng/mL compared to those with values below this level. The authors conclude that higher 25(OH)D was significantly associated with a lower risk of hip FxF in elderly White patients [12], limiting extrapolation to our population and geographic conditions (the term “Non-Hispanic White” is used exactly as reported by the original authors of the referenced study [12]).

Our primary objective was to determine whether lower serum 25(OH)D levels are associated with increased odds of FxF in adult patients hospitalized in the Colombian Caribbean, a region with year-round UV exposure but limited population-based data on vitamin D status. FxF was defined as a low-energy fracture confirmed radiologically at the time of admission. Serum 25(OH)D was analyzed both as a continuous variable and categorized according to established thresholds (deficient, insufficient, sufficient).

We used multivariable logistic regression to adjust for known confounders, including age, sex, BMI, skin phototype (as a determinant of cutaneous vitamin D synthesis), grip strength (as a surrogate for sarcopenia), and comorbidities. Secondary aims included exploring whether skin phototype or other factors modify the association between vitamin D status and FxF and estimating the prevalence of vitamin D insufficiency in this equatorial inpatient population.

## Materials and methods

Study design and setting

We conducted a single-center, cross-sectional, descriptive study at the emergency department of the Hospital Universitario del Caribe (ESE-HUC), a tertiary care facility located in Cartagena, Colombian Caribbean (latitude ~10.3°). Data were collected prospectively between January and March 2024.

Ethical approval and informed consent

This study was conducted in accordance with the Declaration of Helsinki and received approval from the Institutional Ethics Committee of the Faculty of Medicine at the University of Cartagena (approval number: DB-FM-CERT 37 9-08-2023) and was considered a minimum-risk study in accordance with Colombian resolution 8430 of 1993. Written informed consent was obtained from all participants prior to enrollment. Patient confidentiality and data anonymity were preserved throughout the study.

Participants and recruitment

Patients aged ≥60 years were recruited consecutively at admission. The study included 40 patients with FxF and 40 non-fractured (non-FxF) controls, all admitted through the same emergency department. All participants completed the required assessments, and no missing data or losses to follow-up were reported.

Inclusion and exclusion criteria

Eligible participants were hospitalized for either low-energy fractures or acute, non-traumatic medical conditions. Exclusion criteria included high-energy trauma, secondary causes of fracture (e.g., chronic inflammatory rheumatic diseases, long-term glucocorticoid use, morbid obesity, chronic kidney disease, hyperthyroidism, osteomalacia, renal osteodystrophy, type 1 diabetes mellitus, HIV, active cancer, or recent radiotherapy/chemotherapy), atypical bone lesions, or radiographic findings inconsistent with FxF mechanisms. Patients with prior gait limitations or institutionalization were also excluded.

Fragility fracture confirmation

FxF diagnoses were confirmed within 30 days of symptom onset. Hip and distal radius (Colles) fractures presented with pain, deformity, and limited mobility, while vertebral fractures presented with localized pain. All cases were evaluated with radiography on the day of admission and interpreted by board-certified radiologists.

Control group characteristics

Non-FxF controls were admitted for acute conditions unrelated to musculoskeletal trauma, including febrile syndromes, respiratory or urinary tract infections, and cardiovascular events (e.g., arrhythmias, hypertensive crisis). All controls were ambulatory prior to admission and fulfilled the same exclusion criteria.

Clinical and demographic data

Collected variables included age, sex, BMI, smoking status, comorbidities (e.g., hypertension, type 2 diabetes mellitus (T2DM)), glomerular filtration rate (Chronic Kidney Disease Epidemiology Collaboration (CKD-EPI) or microalbuminuria), and medication use (e.g., calcium, vitamin D, osteoporosis therapy). None of the participants had received vitamin D supplementation prior to enrollment.

Sun exposure and skin phototype

Sun exposure was assessed through a structured interview conducted by trained personnel, covering daily exposure time, clothing, and sunscreen use. All participants reported <30 minutes/day of exposure. Skin phototype was classified using the Fitzpatrick scale through visual inspection aided by a standardized reference chart [13]. Both tools are provided in Appendices A-B.

Grip strength measurement

Grip strength was assessed using a Camry EH101 digital dynamometer (Zhongshan Camry Electronic Co., Ltd., Zhongshan, China). Two trials were performed per upper limb (except those affected by Colles fractures), and the highest value was recorded in kilograms. Measurements followed European Working Group on Sarcopenia in Older People 2 (EWGSOP2) recommendations, with patients seated, shoulder adducted, elbow at 90°, and wrist in a neutral position [14]. The dynamometer was calibrated weekly.

Vitamin D measurement

Serum 25(OH)D levels were measured via chemiluminescent microparticle immunoassay (CMIA) using the Abbott Architect i2000SR platform (Abbott Laboratories, Inc., Abbott Park, IL). All samples were drawn before 8:00 a.m. to minimize diurnal variation. The assay's functional sensitivity was 10.0 ng/mL, with intra- and inter-assay coefficients of variation below 10% and 12%, respectively. Quality control procedures followed the manufacturer's guidelines.

Statistical analysis

Quantitative variables were expressed as means ± standard deviations (SD) or medians with interquartile ranges (IQR), depending on distribution. Categorical variables were described as absolute and relative frequencies. Between-group comparisons were performed using Student’s t-test or Mann-Whitney U test for continuous variables and chi-square or Fisher’s exact test for categorical variables.

Multivariable logistic regression was used to identify factors independently associated with FxF. Crude and adjusted odds ratios (ORs) were reported with 95% confidence intervals. Model fit was assessed using the Hosmer-Lemeshow test; multicollinearity was evaluated via variance inflation factors (VIF), and interaction terms were tested for sex and T2DM. Analyses were performed with Epi Info v7.2 (Centers for Disease Control and Prevention (CDC), Atlanta, GA) and STATA version 17.0 (StataCorp LLC, College Station, TX).

A formal sample size calculation was not performed, as this study was conceived as an exploratory, cross-sectional analysis based on the availability of eligible patients during the defined enrollment period. All included patients met the selection criteria and completed the required assessments, with no missing data or loss to follow-up.

## Results

A total of 80 participants were included: 40 participants with FxF diagnosed upon admission to the emergency department of ESE-HUC and 40 non-FxF participants from the same institution who met all inclusion and exclusion criteria except for FxF.

Table 1 shows the overall distribution of the 25(OH)D levels in the study population. Only 32.5% of the study population had normal 25(OH)D levels. In the insufficiency range, 40% of the cases were found in the FxF group and 50% in the non-FxF group (p = 0.057). Deficiency occurred in 25% of the FxF group and 20% of the non-FxF group, and there was no statistical difference between the groups (p = 0.655).

**Table 1:** Distribution of 25(OH)D levels among FxF and non-FxF patients, including categorical classification and serum values (mean ± SD), with p-values for group comparisons. # chi-squared test; $ unpaired Student’s t test; FxF: fragility-fractured, non-FxF: non-fractured; SD: standard deviation

25(OH)D ng/mL	FxF (N = 40)	Non FxF (N = 40)	Test statistic	P
Normal (> 30), n (%)	14 (35)	12 (30)	0.160^#^	0.689
Mean ±SD	37.1 ± 4.4	38.3 ± 7.7	1.17^$^	0.633
Insufficient (20–29.9), n (%)	16 (40)	20 (50)	0.808^#^	0.651
Mean ±SD	23.9 ± 2.69	25.7± 2.77	1.80^$^	0.057
Deficient (< 20), n (%)	10 (25)	8 (20)	0.287^#^	0.592
mean ±SD	13.7 ± 5.3	12.7 ± 4.3	-1.06^$^	0.655

Table 2 summarizes the clinical, demographic, and laboratory characteristics. Eighty-nine percent of FxF participants and 75% of non-FxF participants were female (p = 0.2037). The median age was 73 years in the FxF group (IQR 62-80 years) and 75 years in the non-FxF group (IQR 62-80 years), with no significant difference (p = 0.2037). Median BMI was 22.48 kg/m² in the FxF group (IQR 21.8-23.4 kg/m²) and 22.49 kg/m² in the non-FxF group (IQR 21.89-23.43 kg/m²; p = 0.8208).

**Table 2:** Sociodemographic, clinical, and laboratory variables of cases with FxF and non-FxF # chi-squared test; $ unpaired Student’s t-test; μ-Mann–Whitney U test; BMI: body mass index; FxF: fragility-fractured; non-FxF: non-fractured; OP: osteoporosis; GRF: glomerular rate filtration; T2DM: type 2 diabetes mellitus; IQR: interquartile range

Parameters	FxF N = 40	Non-FxF N = 40	Test statistic	p
Demographics				
Sex, n (%)				
Feminine	36 (90.0)	35 (87.5)	0.125^#^	0.723
Masculine	4 (10.0)	5 (12.5)		
Age, median (IQR)	73 (62–80)	75 (66–84)	668^μ^	0.203
BMI (IQR)	22.4 (21.8–23.4)	22.4 (21.8–23.4)	776.5^μ^	0.82
Clinical features				
Skin phototype n (%)				
I	0 (0.0)	0 (0.0)	--	--
II	13 (32.5)	0 (0.0)	15.222^#^	< 0.0001
III	20 (50.0)	31 (77.5)	6.54^#^	0.01
IV	7(17.5)	9 (22.5)	0.313^#^	0.576
V	0 (0.0)	0 (0.0)	--	--
VI	0 (0.0)	0 (0.0)	--	--
Sun exposure more than 30' /day	0 (0.0)	0 (0.0)	--	--
Use of sunscreen	0 (0.0)	0 (0.0)	--	--
Current smoking	0 (0.0)	0 (0.0)	--	--
Passive smoking	0 (0.0)	0 (0.0)	--	--
Previous skin cancer	0 (0.0)	0 (0.0)	--	--
OP treatment				
Antiresorptives	0 (0.0)	0 (0.0)	--	--
Osteoformers	0 (0.0)	0 (0.0)	--	--
Calcium supplement	0 (0.0)	0 (0.0)	--	--
Vitamin D supplement	0 (0.0)	0 (0.0)	--	--
Clinical findings				
GRF (mL/min/1.73 m²) mean ±SD	66.5 ± 20.4	71.2 ± 19.9	4.69^$^	0.303
Hypertension	12 (30.0)	12 (30.0)	0.00^#^	1
T2DM	12 (30.0)	4 (10.0)	5^#^	0.025
Obesity	0 (0.0)	0 (0.0)	-	-
Serum creatinine mean ±SD	0.93 ± 0.24	0.87 ± 0.24	-0.05^$^	0.318
Hand grip strength, median (IQR)				
Right hand	16 (10–20)	11 (10–13)	503^μ^	0.004
Left hand	16 (10–19)	11 (11–12)	516^μ^	0.006
25(OH)D, ng/mL mean ±SD	26.0 ± 10.0	26.9 ± 10.3	0.89^$^	0.696

Skin phototype III was most frequent, found in 50% of the FxF group and 77% of the non-FxF group (p = 0.0105). Phototype II was more common in the FxF group (13%) than in the non-FxF group (0%, p < 0.0001). Phototype IV was observed in 7% of the FxF group and 9% of the non-FxF group (p = 0.5761). No participants exhibited phototypes V and VI.

No participants had a history of active or passive smoking or skin cancer. All reported < 30 minutes of daily sun exposure and no sunscreen use in the 30 days preceding evaluation.

Most participants had not received osteoporosis treatment or calcium (> 1 g per day) or vitamin D supplementation (800 IU/day). T2DM was present in 12% of the FxF group vs. 4% of the non-FxF group, with a significant difference (p = 0.0253). Hypertension was equally distributed across the groups.

**Figure 1:**
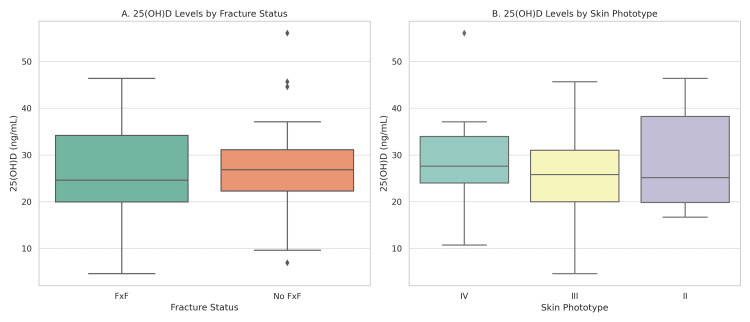
Boxplots of serum 25(OH)D concentrations (A) Comparison of 25(OH)D levels between participants with fragility fractures (FxF) and without fractures (non-FxF); (B) Distribution of 25(OH)D levels across different skin phototypes (II–IV).

Mean GFR was 66.5 ± 20.4 ml/min/1.73 m² in the FxF group and 71.2 ± 19.9 in the non-FxF group (p = 0.3039). The mean hand grip strength was higher in the FxF group (16 kg in both hands; IQR: 10-20 kg and 10-19 kg) than in the non-FxF group (11 kg; IQR: 10-13 kg and 11-12 kg; p = 0.0042 and p = 0.0062). The mean 25(OH)D level was 26.0 ng/mL (SD ± 10.0) in FxF cases and 26.9 ng/mL (SD ± 10.3) in non-FxF cases (p = 0.6961), without statistical significance. Figure 1 illustrates the distribution of 25(OH)D levels according to fracture status and skin phototypes.

Logistic regression (Table 3) showed phototype III was protective against FxF (crude OR 0.29; 95% CI: 0.10-0.76 and adjusted OR 0.27; 95% CI 0.09-0.783, p = 0.015). T2DM increased FxF risk in univariate analysis (OR: 3.86; 95% CI: 1.01-13) but lost significance after adjustment for age, sex, and covariates in multivariate analysis (adjusted OR: 3.3; 95% CI: 0.92-12; p = 0.065). The risk of sarcopenia appeared to be associated with a lower risk of FxF (adjusted OR: 0.04; 95% CI: 0.005-0.333; p=0.03). The logistic regression model showed good calibration according to the Hosmer-Lemeshow test (χ² = 1.275, df = 5, p = 0.937). The pseudo-R² was 0.296 (Cox & Snell) and 0.395 (Nagelkerke), indicating moderate explanatory power. No significant multicollinearity was detected, and none of the interaction terms reached statistical significance, so they were not retained in the final model.

**Table 3:** Logistic regression model with odds ratios (OR) for the risk of fragility fractures (FxF) CI: confidence Interval; T2DM: type 2 diabetes mellitus

Independent variable	Crude OR	95% CI	p	Adjusted OR	95% CI	p
Phototype III skin	0.29	0.110–0.763	0.011	0.279	0.099–0.783	0.015
T2DM	3.86	1.1–13	0.025	3.3	0.92–12	0.065
Vitamin D (< 20 ng/mL)	1.3	0.46–3.8	0.593	1.4	0.45–4.4	0.552
Risk of sarcopenia	0.031	0.004–0.251	0.001	0.04	0.005-0.333	0.03

**Figure 2:**
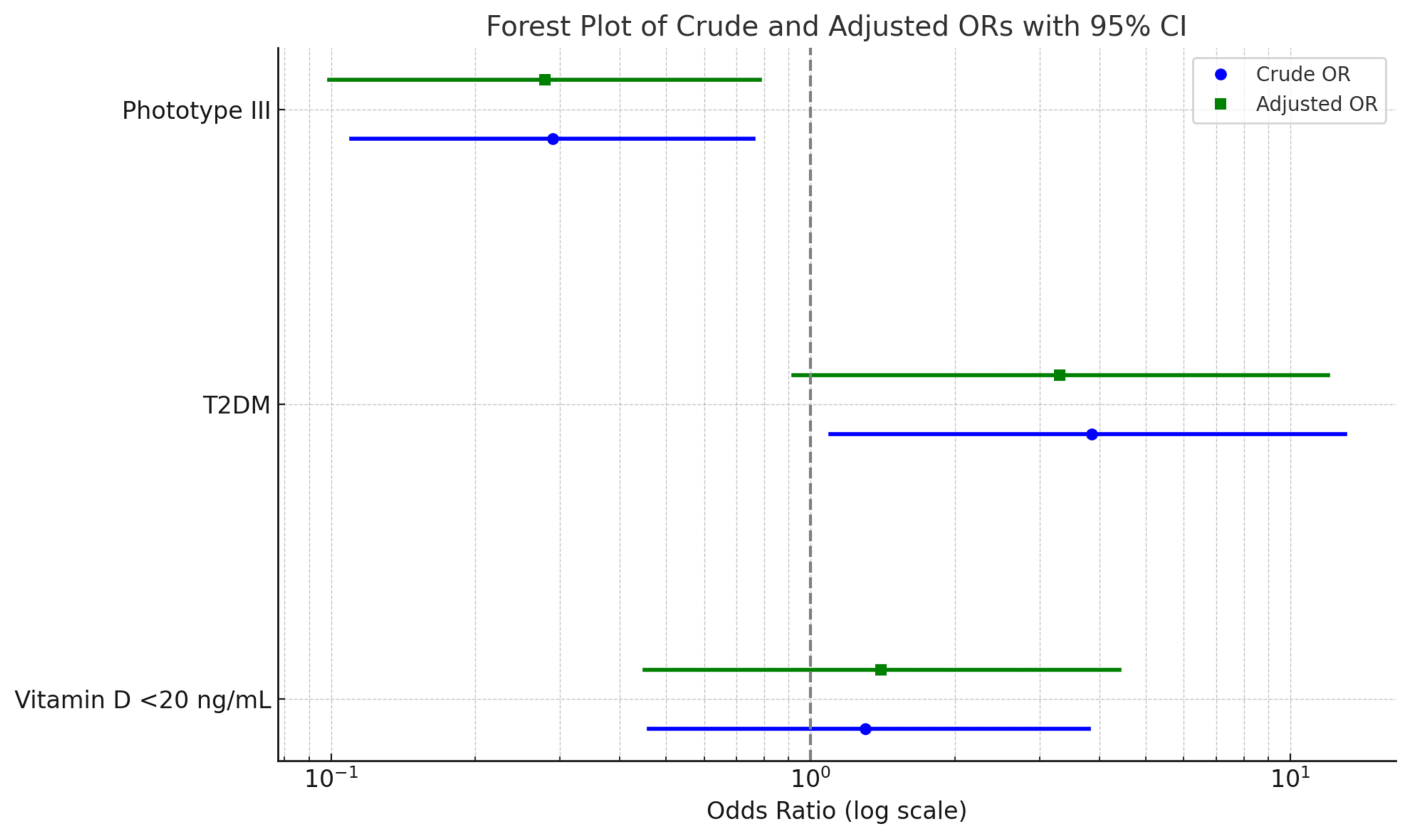
Forest plot of crude and adjusted odds ratios (ORs) (95% confidence interval (CI)) for fragility fractures (FxF) T2DM: type 2 diabetes mellitus

Figure 2 shows the crude and adjusted ORs (with 95% confidence intervals) for key predictors of FxF, including phototype III, T2DM, and vitamin D deficiency. The visual representation highlights that phototype III showed a protective association, while T2DM was associated with increased odds of fracture. ORs are plotted on a logarithmic scale.

The most common fracture site was the hip (57%), with left-side involvement in 47% and right-side involvement in 35%; 5% of FxF cases had prior fractures. The median time from fracture to diagnosis was one day (IQR 0-4 days; Table 4).

**Table 4:** FxF features IQR: interquartile range; SD: standard deviation; FxF: fragility fracture; FxH: fracture of the hip

FxF features	Mean or median (IQR/SD)
Days between fracture and X-ray; median (IQR)	1 (0–4)
Site (SD)	
Vertebral	7 (17.5)
Hip	23 (57.5)
Colles	10 (25.0)
Previous FxF (SD)	2 (5.0)
Previous FxH (SD)	2 (5.0)

## Discussion

This study found that 67.5% of participants had 25(OH)D levels < 30 ng/mL, with only 32.5% reaching sufficiency. Although this represents a lower prevalence compared to global reports, where up to 88% of individuals may fall below this threshold [15], it remains unexpectedly high for a low-latitude region such as the Colombian Caribbean, where year-round sun exposure is presumed to support adequate vitamin D synthesis. For comparison, in Spain, a country with seasonal variability, deficiency rates <20 ng/mL have been reported in 80%-100% of older adults, likely influenced by sun protection practices, low dietary intake, and geographic constraints [16]. Our findings challenge the assumption that equatorial location alone guarantees sufficient 25(OH)D levels. Behavioral and cultural factors, such as limited time outdoors, clothing coverage, and sunscreen use, may reduce effective UVB exposure despite favorable latitude. While comparative data from other tropical regions remain scarce, this pattern underscores the need for more region-specific studies and the potential value of adapting screening and supplementation strategies to local realities.

This study aimed to investigate the association between incident FxF and 25(OH)D in a low-latitude population from a university hospital. The mean 25(OH)D was 26.0 ng/mL (SD ± 10.0) in the FxF group and 26.9 ng/mL (SD ± 10.3) in the non-FxF group, both in the insufficiency range (20-29 ng/mL) [9], with no significant difference between groups (p = 0.69). These values are higher than those reported by Looker [12] and Jaller [17]. A meta-analysis of 20 studies including 41,738 elderly participants with osteoporotic FxF found no significant association between 25(OH)D levels and the overall risk of osteoporotic FxF (p = 0.238; I2 = 21.5%) [18]. Similarly, a genome-wide association study (GWAS) with Mendelian randomization supported these findings and revealed no causal relationship between a predisposition to genetically reduced 25(OH)D levels and FxF risk (OR: 0.84; 95% CI: 0.7-1.02, p = 0.087). In contrast, the same study found that a genetic predisposition to low bone mineral density (BMD) demonstrated a significant causal effect on FxF risk (OR: 1.55; 95% CI: 1.48-1.63; p < 0.001; power: 100%) [19].

These findings differ from those of Looker et al., who identified an association between 25(OH)D levels and hip FxF risk in adults over 65 in the United States. However, their study excluded other ethnicities due to insufficient sample size [12]. In Colombia, Herrera et al. conducted a case-control study at San Ignacio Hospital in Bogota, including 29 FxF cases and 36 controls with no FxF, all of whom had a diagnosis of OP. They found vitamin D deficiency in 35.4% of participants. Cases with FxF had significantly lower 25(OH)D levels (21.5 ± 9.0 vs. 25.8 ± 8.7; p = 0.042), although both groups were within the range of insufficiency (20-29 ng/ml). These findings are similar to ours, though the difference in 25(OH)D levels was statistically significant in their study. However, adjustments were made for potential confounding variables [20].

In Barranquilla, another Caribbean city in Colombia, Jaller et al. carried out a retrospective cross-sectional study in 319 women with a history of FxV, mean age 74.3 years (SD ± 7.2), finding that 54.9% had insufficient 25(OH)D levels. In FxF cases, the mean 25(OH)D level was deficient (19.7 ± 4 ng/mL), whereas it was insufficient in non-FxF cases (25.1 ng/mL ± 8.1 ng/mL; p = 0.005). FxF participants were older (78.9 vs. 73.4 years; p < 0.05) and had lower BMI (23.5 vs. 24.6; p > 0.05). Multivariate analysis was not performed in that study either [16].

In our study, 70% of patients with incident FxF met clinical screening criteria for osteoporosis (OP); however, none had undergone dual-energy X-ray absorptiometry (DEXA) or vitamin D or calcium assessment, and only 5% had a history of prior FxF. Although BMD was not measured via DEXA in this cohort, it is important to clarify that the objective of our study was not to diagnose osteoporosis per se. Rather, we focused on the occurrence of clinically and radiologically confirmed FxF as a surrogate marker of skeletal vulnerability in older adults. Similar findings were reported by Vallejo et al. at the Hospital Universitario San Jorge in Pereira, where only 11.4% of patients meeting screening criteria had been evaluated for OP, and fewer than half received treatment [21]. As in our study, these results highlight the low frequency of screening and intervention for fragility fractures and osteoporosis risk factors.

Another key finding of this study was that neither FxF nor non-FxF participants met international recommendations for vitamin D intake or sun exposure [5], despite these being the primary sources of vitamin D. None of the participants used sunscreen. Vitamin D synthesis and transport are strongly influenced by genetic factors, with single-nucleotide polymorphisms (SNPs) accounting for up to 80% of interindividual variability in plasma vitamin D levels [22], which was beyond the scope of this study.

Skin phototype III was identified as a protective factor against FxF, with no FxF cases observed in individuals with phototypes V and VI. This aligns with findings from Looker et al. and Bao et al., who reported a lower risk of FxF among individuals with darker skin phototypes (e.g., Black, Hispanic, and American Indian) compared to White patients [12, 23]. Thompson et al. suggested that skin photosensitivity, independent of constitutive melanin density, may influence skeletal health by modulating the skin’s response to sun exposure, finding that greater tanning capacity was associated with higher 25(OH)D and BMD levels, without risk of FXF or falls, suggesting that the skin’s response to sun exposure, rather than baseline pigmentation alone, could significantly influence vitamin D metabolism and skeletal health [24]. Notably, our literature review revealed no prior studies in Colombia or Latin America examining the relationship between skin phototype and fragility fracture risk. Therefore, we consider this finding to be novel in the regional context and potentially relevant for populations in low-latitude areas where melanin-related modulation of vitamin D metabolism may play a distinct role.

With regards to the relationship between comorbidities and FxF, T2DM was identified as a potential risk factor in the crude analysis (OR: 3.86; 95% CI: 1.01-13); however, the association did not reach statistical significance in the multivariate model after adjusting for age, sex, and other comorbidities (adjusted OR: 3.3; 95% CI: 0.92-12.0; p = 0.065). Although not statistically significant, the observed trend suggests a possible association that may not have been detected due to limited statistical power, raising the possibility of a type II error. Larger studies are warranted to further explore this relationship.

T2DM is typically characterized by normal or even elevated BMD, yet paradoxically associated with increased fragility fracture risk due to mechanisms such as greater cortical porosity, accumulation of advanced glycation end-products, impaired bone biomechanics, and elevated sclerostin levels that inhibit bone formation [25]. The lack of significance in our adjusted model may reflect sample size limitations that reduced power to detect a true effect. Moreover, large-scale GWAS, including the meta-analysis by Trajanoska et al., did not show causal associations between fragility fractures and traditional risk factors such as rheumatoid arthritis (OR: 1.00; 95% CI: 1.00-1.02; p = 0.14), ischemic heart disease (OR: 1.00; 95% CI: 0.90-1.02; p = 0.76), or T2DM (OR: 0.84; 95% CI: 0.99-1.01; p = 0.37) [19]. While Mendelian randomization studies provide valuable causal insights, our study was observational and did not include genetic data; these external findings are referenced to contextualize our results and do not imply causality within our cohort.

Handgrip strength, used in this study as an indirect measure of sarcopenia, was higher in FxF cases than in non-FxF controls. According to the 2019 EWGSOP2 guidelines, probable sarcopenia is defined as <27 kg in men and <16 kg in women [14]. In our cohort, median grip strength in men with FxF fell within the sarcopenic range, while in women it was at the lower limit of normal. In contrast, grip strength in the non-FxF group was lower in both sexes. This counterintuitive result may be explained by the clinical characteristics of the control group, which included patients admitted for acute illness, many with multimorbidity and functional decline. These conditions may have reduced grip strength independently of chronic sarcopenia, leading to an apparent paradox in the bivariate analysis. Moreover, logistic regression revealed that lower grip strength was associated with reduced odds of FxF. This finding should be interpreted cautiously, as it may reflect a selection artifact due to the hospital-based nature of the sample. Both sarcopenia and FxF increase the likelihood of hospitalization, which could introduce collider bias, artificially distorting their association. This methodological limitation is important when analyzing complex interactions in non-randomized, inpatient populations. Although handgrip strength is not a direct determinant of BMD, it is strongly associated with overall muscle function and fall risk, key contributors to FxF, especially in older adults [26]. A Mendelian randomization study by Song et al. further supported the role of muscle strength as a contributor to FxF risk, independent of BMD [27]. Thus, the higher grip strength observed in FxF cases in our study likely reflects differences in baseline functionality and health status between the two hospital-based groups, rather than a true protective effect. Further studies in community-dwelling populations are needed to explore this relationship in settings free from hospitalization-related selection bias. Additionally, the proposed explanation regarding the role of multimorbidity in the control group remains speculative and should be validated in future prospective cohorts.

Among the strengths of this study is its prospective design, which allowed for the timely collection of serum 25(OH)D levels in participants with incident FXF, thereby reducing the potential for sampling and recall bias. Furthermore, the inclusion of a wide range of laboratory variables, skin phototype, comorbidities, and hand grip strength provided a comprehensive view of factors associated with FxF in a low-latitude setting. Handgrip strength evaluation, although ultimately excluded from the final multivariate analysis, served as a valuable surrogate for sarcopenia and a potential confounder.

Limitations

This study has several limitations that should be considered when interpreting the findings. First, both FxF and non-FxF participants were recruited from a hospital emergency setting, meaning that all individuals were acutely ill at the time of assessment. Acute illness may transiently lower circulating 25(OH)D levels through inflammatory pathways, such as IL-6 and TNF-α activation, which can affect vitamin D metabolism and reduce measurable levels during systemic illness [28,29]. This may have contributed to the generalized insufficiency observed in our cohort and limits generalizability to healthier, community-dwelling populations. The cross-sectional design precludes causal inference and does not establish temporality between vitamin D status and FxF. Although serum 25(OH)D was collected prospectively upon admission, potential acute-phase responses may confound interpretation of baseline levels.

Although fragility fractures were radiologically confirmed, BMD was not assessed via DEXA. Nonetheless, it is important to clarify that the objective of this study was not to diagnose osteoporosis but to examine the presence of FxF as a clinically relevant outcome in older adults. The modest sample size (n=80) may have limited statistical power to detect moderate associations or perform stratified analyses, raising the potential for type II errors, particularly in the analysis of comorbidities such as T2DM. Although controls were selected using the same inclusion and exclusion criteria, no formal matching or randomization strategy was applied. This may introduce selection bias, and residual confounding cannot be excluded due to unmeasured variables such as dietary intake, physical activity, socioeconomic status, and BMD.

Finally, while measurement procedures were standardized, some protocols may lack external reproducibility. Skin phototype was assessed visually using a standardized chart, sun exposure was self-reported, and sarcopenia was evaluated solely via grip strength without complementary assessments of muscle mass or function.

## Conclusions

Serum 25(OH)D levels were insufficient in both FxF and non-FxF groups, although the overall deficit was lower than that reported in populations at higher latitudes. Notably, none of the participants had undergone prior screening for osteoporosis or vitamin D assessment, despite meeting clinical criteria for evaluation. Only 5% of those with FxF had a history of previous fractures. In addition, no participant met international recommendations for vitamin D intake or sun exposure, and none were receiving supplementation at the time of the study. Skin phototype III or higher was identified as a protective factor against FxF, in line with findings from studies conducted in the United States. However, similar research in Latin America or other low-latitude populations is lacking. The observed association may reflect differences in skin response to UVB exposure, but further investigation is needed to clarify this relationship.

The inclusion of acutely ill, hospitalized patients may help explain the high prevalence of 25(OH)D insufficiency and the paradoxically lower grip strength in non-FxF participants, which could be influenced by greater multimorbidity. These results highlight the complexity of vitamin D metabolism and its interaction with functional status in clinical settings. Therefore, further research is warranted to explore the impact of genetic variants, particularly those affecting vitamin D metabolism such as CYP2R1, DHCR7, GC, and CYP24A1, as well as comorbidities like T2DM, on vitamin D status and fracture risk in diverse populations. Further studies in low-latitude, community-based populations are needed to validate these findings and inform the development of targeted screening and prevention strategies tailored to regional and biological contexts.

## Figures and Tables

**Figure 3:**
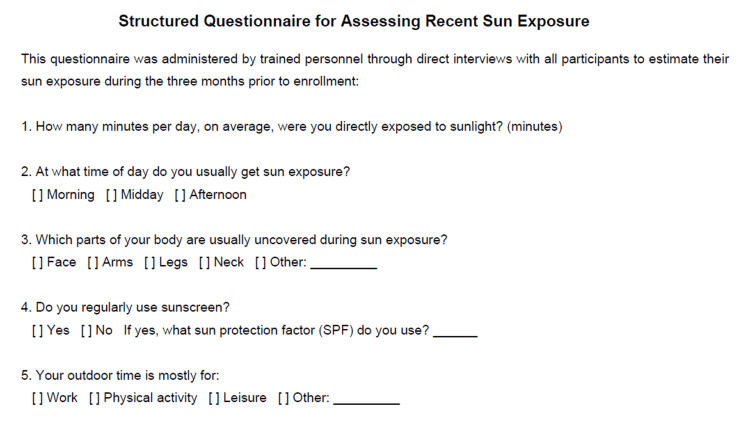
Sun exposure assessment

**Figure 4:**
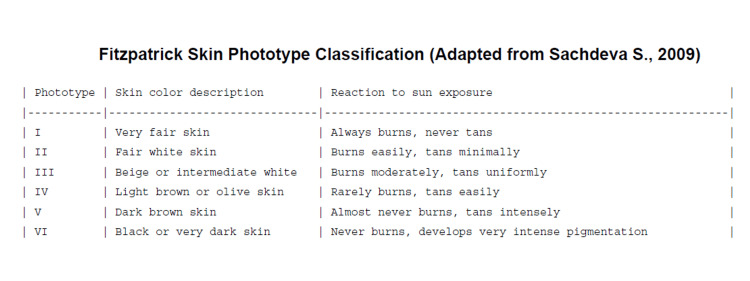
Skin phototype classification Source: [13]

## Appendices

Appendix A 

Appendix B 
